# Motion correction in CBCT imaging using gate‐less model‐based reconstruction of non‐rigid motion and images

**DOI:** 10.1002/mp.70070

**Published:** 2025-10-09

**Authors:** Ethan Waterink, Rodrigo José Santo, Cornelis A. T. van den Berg, Alessandro Sbrizzi, Casper Beijst

**Affiliations:** ^1^ Department of Radiotherapy Utrecht University and University Medical Center Utrecht Utrecht The Netherlands; ^2^ Computational Imaging Group Center for Image Sciences and University Medical Center Utrecht Utrecht The Netherlands

**Keywords:** CBCT motion correction, gate‐less motion reconstruction, model‐based non‐rigid motion

## Abstract

**Background:**

Cone beam computed tomography (CBCT) plays a crucial role in verifying patient positioning during radiotherapy. However, CBCT images are often blurred by motion originating from breathing, bowel motion, and/or repositioning. Conventional methods often employ gating techniques to mitigate motion artifacts by assuming periodicity, which is restricted to respiratory motion. However, this does not resolve a‐periodic motion such as irregular breathing.

**Purpose:**

The purpose of this study is to estimate and correct for both periodic motion and irregular motion. We present a novel method for gate‐less reconstruction of motion and images (CBCT‐MOTUS), thereby estimating and correcting for all non‐rigid 3D motion at high temporal frequency (per‐projection temporal resolution of 182 ms).

**Methods:**

Starting from a motion‐corrupted image, we perform the reconstruction process by alternating between a motion estimation step and an image correction step. Motion estimation is model‐based, that is, it is performed directly in projection space by comparing acquired projections to simulated projections that take motion‐fields into account. The optimization process benefits from assumptions, including (i) compressing the motion‐fields using B‐spline parameterization to reduce the number of parameters to estimate, (ii) exploiting spatio‐temporal correlation of motion using a low‐rank motion model, and (iii) enforcing smoothness using spatial regularization to include a priori knowledge on motion‐fields. High temporal resolution (182 ms for this scanner) is achieved as motion‐fields are estimated for each acquired gantry angle. The method is validated in silico, on phantoms and on clinical in vivo acquisitions.

**Results:**

The framework can estimate and correct irregular and periodic motion with high temporal resolution (per‐projection temporal resolution of 182 ms). The motion‐corrected images show improved image features for all experiments with reduction of motion artifacts, such as deblurring on organ interfaces.

**Conclusions:**

We have developed and validated a gate‐less reconstruction method (CBCT‐MOTUS) for model‐based motion estimation and correction in CBCT imaging for any kind of motion. High temporal resolution (per‐projection temporal resolution of 182 ms) is achieved by exploiting the smoothness and compressibility of motion using a low‐rank B‐spline motion model, enabling the correction for non‐rigid irregular and periodic motion. These proof‐of‐principle results warrant further clinical validation.

## 1 INTRODUCTION

Radiotherapy is the cornerstone of many cancer treatments. Anatomically accurate images acquired prior to radiotherapy are essential for accurate treatments, especially when smaller margins are used for daily adjustments, referred to as adaptive radiotherapy.^1^ Cone‐beam computed tomography (CBCT) is often used for position verification prior to radiotherapy. However, a major challenge in acquiring high‐quality CBCT images is motion from breathing, bowel motion, and/or repositioning, especially a‐periodic and irregular motion.

Conventional methods often employ gating or sorting techniques to mitigate motion artifacts in (CB)CT. They assume periodicity of motion to reconstruct different phases separately, from which the deformation vector fields are subsequently estimated by non‐rigid registration.^2^ Gating has been shown to improve target localization discrepancy and organ visibility.^3^ However, gating does not solve irregular, a‐periodic motion such as dysfunctional breathing, resulting in images with inherent blurring. Irregular motion can also lead to misalignment artifacts and disconnected anatomy.^4^ Moreover, registration in image space is affected by reconstruction artifacts and data sparsity for individual frames/gates.^5^

4D CBCT scans have longer acquisition time to collect more data for higher‐quality gating or sorting. Together with a surrogate signal, data can be sorted into phases for reconstruction.^6^ A surrogate‐driven approach for model fitting of respiratory motion has been deployed by extracting respiration surrogate signals from the projection data by the intensity analysis method.^7^ Motion compensation in cardiac imaging can be performed with an ECG surrogate signal.^8^ Navigators are used to track patient motion, sometimes internally^9^ or externally.^10^ These approaches require longer acquisition times and increase in delivered radiation, or use (external) surrogate signals.

Artificial intelligence (AI) has been employed as pre‐ and/or post‐processing steps for motion estimation and correction,^19^ or as part of the reconstruction process.^20^ Real‐time CBCT imaging has been implemented with deep‐learning by reconstructing a dynamic CBCT from a pre‐treatment CBCT scan,^21^ and by capturing spatiotemporal correlation in the projections using PCA coefficient estimation.^22^ Neural networks have been trained to map motion‐corrupted projections to motion‐free projections by “freezing” the patient in a certain motion phase from 4D CT scans.^23^ Deep learning networks are generally black box methods and do not provide the same understanding as model‐based approaches. However, hybrid methods combine deep learning with models for better understanding. Deep‐autofocus methods leverage convolutional neural networks to estimate motion‐fields, which are used to compensate for motion in analytical reconstruction.^24^, ^25^, ^26^

Joint reconstruction methods simultaneously perform reconstruction of motion and images using the full projection data, resulting into less noise. The motion optimization problem has been solved in projection space with a derivative free approach using the Nelder–Mead algorithm,^11^ or with a gradient‐based optimization algorithm and generalized derivatives of cone beam backprojection operator.^12^ Recently, motion compensation has been achieved via a surrogate‐optimized respiratory motion from unsorted projection data.^13^ Additionally, diffeomorphic motion models are used to incorporate fundamental physical properties of organ motion.^14^ Motion detection algorithms have been used to isolate motion‐free projections from which a regularized reference reconstruction is made, after which motion is estimated.^15^ Other approaches for irregular motion use the autofocus concept to estimate motion by maximizing a sharpness metric computed on the reconstructed image (e.g., image entropy, total variation or a gradient‐based metric).^16^, ^17^, ^18^ Most previously proposed joint reconstruction methods only perform rigid motion estimation.

In this work, we present a novel method for gate‐less model‐based reconstruction of motion and images in CBCT imaging, thereby estimating and correcting for all non‐rigid 3D motion at high temporal frequency (per‐projection temporal resolution of 182 ms). We assume that motion can be parameterized by parsimonious models. Therefore, we postulate that the motion estimation problem can be optimized by (i) compressing the motion‐fields using B‐spline parameterization to reduce the number of parameters to estimate, (ii) exploiting spatio‐temporal correlation of motion using a low‐rank motion model, and (iii) enforcing smoothness using spatial regularization to include a priori knowledge on motion‐fields. Starting from a motion‐corrupted image, we resolve for motion by alternating between a motion estimation and an image correction step. Motion estimation is performed directly in projection space (model‐based) by comparing acquired projections to simulated projections that take motion‐fields into account. High temporal resolution is achieved as motion‐fields are estimated for each acquired gantry angle. We draw inspiration from the MRI framework Model‐based Reconstruction of MOTion from Undersampled Signals (MR‐MOTUS)^27^ and subsequent developments^28^, ^29^ for our CBCT setting, hence the name CBCT‐MOTUS. Proof‐of‐principle tests of CBCT‐MOTUS are conducted on in silico, phantom and clinical acquisitions.

## 2 THEORY

**FIGURE 1:**
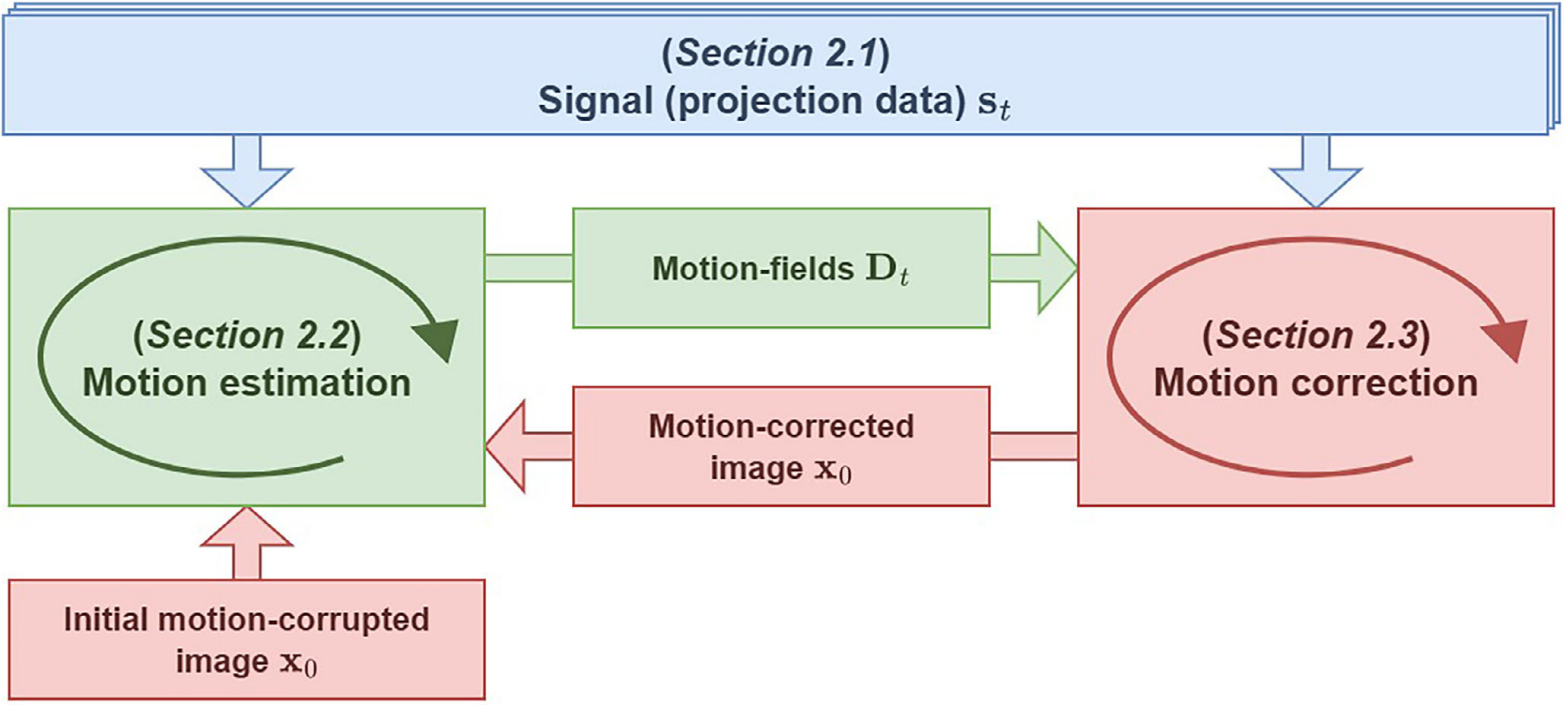
Schematic overview of the CBCT‐MOTUS framework: alternate between motion estimation and correction. Motion estimation is performed directly in projection space (model‐based) using the acquired signal (projection data) and simulated projections of motion‐corrupted/corrected images. Motion correction is performed with the estimated motion‐fields. CBCT, cone beam computed tomography.

The CBCT‐MOTUS framework performs the reconstruction process by alternating between motion estimation and correction (Figure 1), starting from a motion‐corrupted image. Motion estimation is performed directly in projection space (model‐based) by comparing the acquired signal (Section 2.1) to simulated projections that take motion‐fields into account (Section 2.2). Motion correction is performed with the estimated motion‐fields (Section 2.3). Motion estimation and correction are subsequently iteratively performed with, respectively, the motion‐corrected images and estimated motion‐fields. We will refer to one step of motion estimation and correction as an *alternation*. For the overall CBCT‐MOTUS framework, we refer the reader to Algorithm 1.

ALGORITHM 1 **The CBCT‐MOTUS framework for joint reconstruction of motion estimation and correction.**

**Table:** 

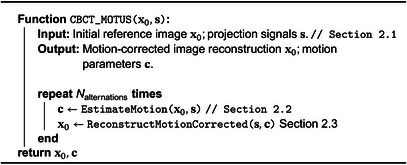

### 2.1 Signal

Let $x_{t}\left[r\right]$ denote the densities of a deforming object at time t over a uniform spatial grid r. A CBCT system rotates an x‐ray source and imaging sensors (detectors) around the object and measures the photon intensity after attenuation through the object. The projection signal can be modeled as

(1) $$\begin{array}{cc} s_{t} & =s_{0}\exp\left(-\int_{L_{t}}x_{t}dr\right)\\ & =s_{0}\exp\left(-y_{t}\right), \end{array}$$

where $s_{0}$ is the source intensity, $L_{t}$ are the lines from source to detectors, and $y_{t}$ the line integrals. The modeling is mono‐energetic and does not take into consideration non‐idealities of the imaging chain such as scattering and noise. Line integrals are modeled through the system matrix $A_{t}={\left[a_{ij}\right]}_{t}$, specifying the contribution of voxel j to detector i, giving

(2) $$y_{t}=A_{t}x_{t}.$$

Then, let $D_{t}$ be the motion‐field that deforms $x_{0}$ to $x_{t}$:

(3) $$\begin{array}{cc} x_{t}\left[r\right] & =x_{0}\left[D_{t}\left(r\right)\right]\\ & =x_{0}\left[r+\delta_{t}\left(r\right)\right], \end{array}$$

with displacement function $\delta_{t}$ to sample $x_{0}$. We will refer to $x_{0}$ as the *reference image*, $s_{t}$ as the *projection signal*, and $y_{t}$ as the *integral signal*. The signal is acquired at high frequency from beginning to end of the acquisition, which we will refer to as *time‐resolved*. The reference image is an image reconstruction in an arbitrary motion state and is updated in each alternation. Since the motion is time‐resolved, any time frame can be used as the reference motion state and no separate stationary scan is necessary for registration. For the first alternation, a motion‐corrupted image, that is, without any motion compensation. An explicit relation between the reference image, motion‐fields, and the projection/integral signal of the deforming object can be obtained by substituting (3) in (2) and rewriting (1) to obtain the signal model:

(4) $$y_{t}=-\ln\left(\frac{s_{t}}{s_{0}}\right)=A_{t}x_{0}\left[D_{t}\left(r\right)\right].$$

This relation ensures that the signal model is linear rather than exponential.

### 2.2 Motion estimation

Motion estimation is performed directly in projection space by minimization of a model‐based functional. High temporal resolution is achieved as motion‐fields are estimated for each acquired gantry angle. However, motion estimation for all three components is challenging due to limited angular information per time frame. The optimization process benefits from a priori assumptions that the motion‐fields can be compressed using B‐spline parameterization to reduce the number of parameters to estimate (Section 2.2.1), the spatio‐temporal correlation of motion can be exploited using a low‐rank motion model (Section 2.2.2), and that smoothness can be enforced using spatial regularization to include a priori knowledge on motion‐fields (Section 2.2.3).

Note that (4) explicitly connects the integral signal to a reference object through non‐rigid motion‐fields $D_{t}$. That is, if reference image $x_{0}$ and integral signal $y_{t}$ are available, the motion can be estimated by solving the inverse problem corresponding to (4). We parameterize the motion‐fields with a lower‐dimensional basis using basis coefficients c to reduce the number of parameters. Writing (4) in operator form

$$\begin{array}{cc} y_{t} & =F_{t}\left(c|x_{0}\right)\\ & =A_{t}x_{0}\left[D_{t}\left(r|c\right)\right], \end{array}$$

where $F_{t}$ is the model that performs image‐domain deformations and forward projections, we estimate motion‐fields by solving the following minimization problem (Section 2.2.4):

(5) $$\underset{c}{\arg\min}\sum_{t=1}^{T}{∥F_{t}\left(c|x_{0}\right)-y_{t}∥}_{2}^{2}+\lambda R\left(D_{t}\left(r|c\right)\right),$$

where T is the number of time frames, R is a spatial regularizer that models a priori knowledge on motion‐fields, and $\lambda \in R^{+}$ controls the strength of the regularization. Note that there is no explicit modeling of noise in the projection data.

#### 2.2.1 Motion compression

We assume that motion is spatially smooth, which has been shown to be highly compressible.^27^ Accordingly, we can represent the motion in a lower‐dimensional basis to compress it and reduce the number of parameters to optimize. We obtain smooth non‐rigid motion‐fields by defining the spatial parametrization as uniformly spaced control points of cubic B‐splines. The number of control points per dimension affects the complexity of motion and local smoothness.

#### 2.2.2 Motion model

Patient motion contains high spatio‐temporal correlation between subsequent time frames. That is, motion is spatially and temporally smooth. Respiratory motion, for example, has a semi‐periodic nature. We exploit this correlation with a low‐rank motion model^30^ to separate the motion into spatial and temporal components. This further reduces the number of parameters to optimize for. A single angle only contains 2D information, making it more difficult to estimate 3D motion‐fields. Temporal splines allow for correlation between frames and thus enable estimating 3D motion‐fields from 2D images. Several components can be combined to account for different types of motion, including irregular motion. The modeled motion is defined by scaling spatial components with temporal ones:

(6) Dt(r∣c)=r+∑c=1Ncompbx(r)ccxby(r)ccybz(r)ccz︸σc(r)bτ(t)ccτ︸τc(t)

where spatial coefficients $c^{x,y,z}$ are expanded by 3D B‐spline basis functions $b^{x,y,z}$ evaluated on grid r, and temporal coefficients $c^{\tau }$ are expanded by 1D B‐spline basis function $b^{\tau }$ evaluated at time t. That is, for each spatio‐temporal component c, the spatial component σ is scaled by temporal component τ. The final motion‐field is constructed by adding all $N_{\mathrm{comp}}$ spatio‐temporal components, which equals the rank of the model.

#### 2.2.3 Motion regularization

Regularization of the motion‐fields is necessary due to the limited angular information for each time frame. Therefore, trying to estimate motion merely from data consistency in projection space is an ill‐posed problem. The spatial regularizer models prior information on the motion‐fields to encourage realistic motion. In particular, we apply the total variation (TV)^31^ on the Jacobian of the motion‐fields for regularizer R:

$$R\left(D_{t}\left(r|c\right)\right)=\sqrt{\sum_{p\in \{x,y,z\}}{\left(\sum_{d\in \{x,y,z\}}{\left\|\frac{\partial D_{t}^{p}\left(r|c\right)}{\partial d}\right\|}_{2}\right)}^{2}},$$

where $\frac{\partial D^{p}}{\partial d}$ is the gradient in the d‐direction of the pth dimension of the motion‐field. This expression considers all components of the displacement gradients jointly. The gradients of the motion‐fields can be computed analytically from (6) using B‐spline derivatives or numerically by finite differences.

#### 2.2.4 Motion optimization

The minimization problem of (5) is performed directly in projection space by minimizing the difference between the acquired signal and simulated projections created from deformed reference images, with respect to motion parameters c. The deformations are controlled by the motion compression, low‐rank motion model and spatial motion regularization.

Derivative‐based algorithms can be used for solving this minimization problem. Specifically, we apply gradient descent to find motion parameters c by rephrasing (5) and minimizing loss function

(7) $$L\left(c\right)=\sum_{t=1}^{T}{∥F_{t}\left(c|x_{0}\right)-y_{t}∥}_{2}^{2}+\lambda R\left(D_{t}\left(r|c\right)\right).$$

We use PyTorch's automatic differentiation (autograd)^32^ functionality to automatically compute derivatives. It tracks all operations performed on motion parameters c and computes the gradients $\frac{\partial L}{\partial c}$ by applying backpropagation once (7) has been evaluated. We explicitly define the backward projection $A_{t}^{T}$ as the backward step for the line integrals. We use the NAdam optimizer^33^ as the gradient descent algorithm with $N_{\mathrm{epochs}}$ epochs and learning rate γ. The motion parameters c are randomly initialized. For the pseudocode of the algorithm we refer the reader to Algorithm 2.

ALGORITHM 2 **Motion estimation: gate‐less model‐based non‐rigid motion estimation algorithm.**

**Table:** 

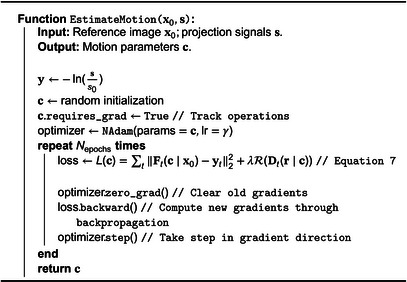

### 2.3 Motion correction

Motion correction is performed to mitigate related artifacts by reconstructing an image to a single reference position using the motion‐fields obtained through the methods of Section 2.2. Image reconstruction requires projection signals over a range of gantry angles. Concatenating the system matrices and integral signals of all time frames, we get

(8) $$\underset{x}{\arg\min}∥Ax-y∥.$$

In order to compute x in (8), we solve a weighted‐least squares problem using the simultaneous iterative reconstruction technique (SIRT),^34^ given by

(9) $$\begin{array}{cc} x^{\left(n+1\right)} & =x^{\left(n\right)}+CA^{T}R\left(y-Ax^{\left(n\right)}\right)\\ & =x^{\left(n\right)}+C\sum_{t}A_{t}^{T}R_{t}\left(y_{t}-A_{t}x^{\left(n\right)}\right), \end{array}$$

where $C=\frac{1}{\sum_{t=1}^{T}A_{t}^{T}1_{P}}$ and $R_{t}=\frac{1}{A_{t}1_{I}}$ are normalizations, and $1_{P}$ and $1_{I}$ images of ones in projection and image space, respectively. This method iteratively updates the current guess by backprojection of the error between the measured data and simulated projections for all time frames.

We incorporate motion correction by first deforming the current guess to match the motion state of $y_{t}$, assuming that the former is in the reference position. Let $W_{t}$ be the deformation operation corresponding to (3):

(10) $$\begin{array}{cc} x_{t}\left[r\right] & =x_{0}\left[D_{t}\left(r\right)\right]\\ & =W_{t}\left\{x_{0}\right\}\left[r\right], \end{array}$$

and let $D_{t}^{-1}$ be the inverse motion‐field that deforms $x_{t}$ to $x_{0}$ with corresponding deformation operator $W_{t}^{-1}$:

(11) $$\begin{array}{cc} x_{0}\left[r\right] & =x_{t}\left[D_{t}^{-1}\left(r\right)\right]\\ & =x_{t}\left[r+\delta_{t}^{-1}\left(r\right)\right]\\ & =W_{t}^{-1}\left\{x_{t}\right\}\left[r\right], \end{array}$$

with $\delta_{t}^{-1}$ the inverse displacement function of $\delta_{t}$ such that

(12) $$\begin{array}{cc} D_{t}^{-1}\left(D_{t}\left(r\right)\right) & =r=D_{t}\left(D_{t}^{-1}\left(r\right)\right),\\ W_{t}^{-1}\left\{W_{t}\left\{x\right\}\right\} & =x=W_{t}\left\{W_{t}^{-1}\left\{x\right\}\right\}. \end{array}$$

Assuming that the current guess $x^{\left(n\right)}$ is in the reference position, we must first deform it with $W_{t}$ to match the motion state of $y_{t}$. After computing the error and backprojection, the result is warped back with $W_{t}^{-1}$ to the reference position to perform motion correction. Moreover, a non‐negativity constraint is applied. For the pseudocode of the algorithm we refer the reader to Algorithm 3.

ALGORITHM 3 **Motion correction: motion‐corrected image reconstruction algorithm.**

**Table:** 

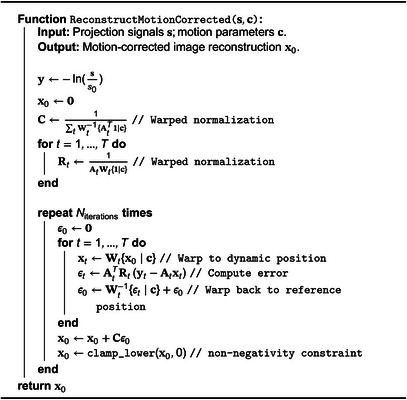

In general, the exact inverse displacement function $\delta_{t}^{-1}$ does not exist. Instead, we make use of adjoint pull and push operations, which, respectively, sample and splat an image with respect to a motion‐field. Specifically, we define

(13) $$\begin{array}{cc} W_{t}\left\{x\right\} & =\mathrm{pull}(x,D_{t}\left(r\right)),\\ W_{t}^{-1}\left\{x\right\} & =\mathrm{push}\left(x,D_{t}\left(r\right)\right)/\mathrm{push}\left(1,D_{t}\left(r\right)\right), \end{array}$$

where we normalized the latter to account for the number of voxels splatting into each voxel.

## 3 METHODS

The following data were acquired in three different experiments:

1. **In silico** allows for validation against ground truth reference images and motion‐fields,
2. **Phantom acquisition** validates against static images and ground truth motion‐fields,
3. **Clinical acquisitions** are patient data providing more complex motion‐fields.

We evaluated the performance of the CBCT‐MOTUS framework for all experiments by comparing the initial motion‐corrupted reference with the motion‐corrected image, and with the use of line profiles. Moreover, we quantified the in silico experiment by computing the structural similarity index measure (SSIM) between static and motion‐corrupted/corrected images over the field of view (FOV) volume, and by (the inverse of) the spatial resolution $w_{\mathrm{ESF}}$ estimated as the width of the edge spread function (ESF) fitted on the line profiles.^26^

### 3.1 In silico: XCAT phantom

In the first experiment, the digital anthropomorphic XCAT phantom^35^ was utilized with varying amplitudes and frequencies of respiration for realistic physiological motion with an average respiratory period of 5 s. We defined the ground truth motion using the initial reference image and motion‐fields to create each dynamic. The projection signals were simulated by forward projecting each dynamic, using cone geometry and gantry angles from a clinical scan. Expanding on (1), the simulated signal becomes

$$s_{t}=\mathrm{Poisson}\left(s_{0}\exp\left(-y_{t}\right)\right)+\mathrm{Normal}\left(0,\sigma_{\mathrm{electronic}}^{2}\right),$$

where Poisson adds Poisson noise to the projection signal to represent X‐ray quantum noise, and Normal adds electronic background noise with variance $\sigma_{\mathrm{electronic}}^{2}$ to test the framework's robustness to noise. We set the source intensity $s_{0}=1.0\cdot 10^{5}$ and $\sigma_{\mathrm{electronic}}^{2}=10$.^22^

Moreover, we quantitatively evaluated the performance of the motion estimation method by re‐running it using a motion‐ and artifact‐free reference image (i.e., the XCAT ground truth image) for $x_{0}$ in (5) instead of the motion‐corrupted image. This provides validation that using motion‐corrupted/corrected images as surrogates for motion‐free reference images is effective.

### 3.2 Phantom acquisition

**FIGURE 2:**
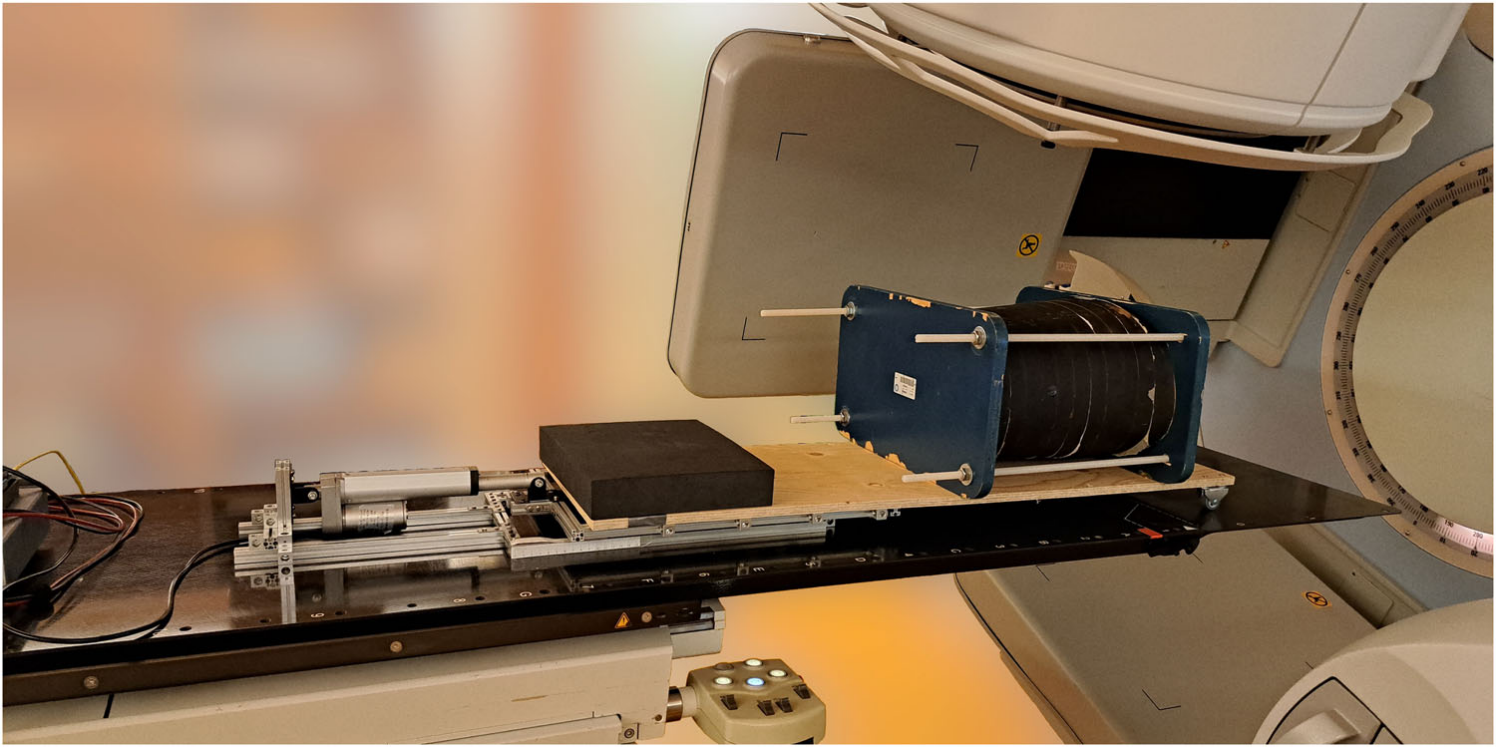
Phantom acquisition setup with part of an Alderson radiation therapy torso phantom placed on a programmable moving platform.

With the second experiment, part of an Alderson radiation therapy (ART) torso phantom was placed on an in‐house developed moving platform,^36^ constructed with a DC electronic motor and programmable through Arduino, inside the Elekta XVI system (Elekta AB, Stockholm, Sweden)—with 512×512 pixels, 0.8 mm spacing, and 1536 mm SDD—for data acquisition (Figure 2). This phantom is made up from individual slices with gaps/paper between them. We applied 1D transverse motion mimicking a respiratory pattern with different amplitudes and slightly different periods. As the phantom was larger than the transverse FOV, motion was also present outside the FOV. Similar to the in silico experiment, the ground truth motion is known and can be used for comparison. Moreover, we made a scan of the static phantom for reference.

### 3.3 Clinical acquisitions

For the third experiment, we utilized raw patient data from clinical CBCT examinations acquired for position verification prior to radiotherapy at the University Medical Center Utrecht. As we used clinically obtained anonymized data, our medical ethics committee waived the requirement for written informed parental consent for participation in the study. We used the data of four patients: patient A (lung cancer), patient B (lung cancer), patient C (bone metastases), and patient D (lung cancer).

### 3.4 Convergence

The CBCT‐MOTUS framework alternates between motion estimation and image correction steps until a maximum number of alternations or convergence has been reached. We define convergence for our framework when the motion estimation no longer improves (determined by visual inspection).

### 3.5 General settings

The acquisition time for all experiments was 29 s with 182 ms between consecutive projections, creating 160 frames in total, covering $180^{\circ }$. The temporal resolution of the motion is also 182 ms as we estimate 3D motion‐fields for each projection. The source intensity $s_{0}$ was estimated for the phantom and clinical experiments from their projection signals by computing the mean value of an area of background pixels (i.e., where there was no object between source and detector). We extended the reconstruction domain to prevent reconstruction artifacts in the SIRT reconstructions to account for the fact that the patient is not fully inside the scanner's FOV. The regularization strength was determined empirically such that motion is relatively smooth while ensuring sufficient data consistency. See Table 1 for all relevant parameter settings of the motion estimation and correction.

**TABLE 1:** Details of the experiments: settings for image reconstruction, motion model, and motion estimation.

Parameter	In silico	Phantom acquisition	Clinical acquisitions
Resolution	256×256×256	264×270×270	264×270×270
Voxel size (mm)	1.2×1.2×1.2	1.15×1.22×1.22	1.15×1.22×1.22
Number of reconstruction iterations $N_{\mathrm{iterations}}$	200	200	200
Spatial spline control points per dimension	32	7	32
Temporal spline control points	2.0 $s^{-1}$	2.0 $s^{-1}$	3.0 $s^{-1}$
Number of spatio‐temporal components $N_{\mathrm{comp}}$	1	1	3
Learning rate γ	$1.0\cdot 10^{-1}$	$1.0\cdot 10^{-1}$	$1.0\cdot 10^{-1}$
Spatial regularization coefficient λ	$1.0\cdot 10^{-6}$	$1.0\cdot 10^{-6}$	$1.0\cdot 10^{-6}$
Number of motion estimation epochs $N_{\mathrm{epochs}}$	100	100	100
Maximum number of alternations $N_{\mathrm{alternations}}$	50	50	20

The scanner was processed with the ASTRA Toolbox^37^, ^38^ on the NVIDIA Quadro P6000 graphical processing unit (GPU) using PyTorch and the tomosipo^39^ library for 3D tomography. Warped volumes were resampled on a grid using tri‐linear interpolation.

## 4 RESULTS

### 4.1 In silico: XCAT phantom

**FIGURE 3:**
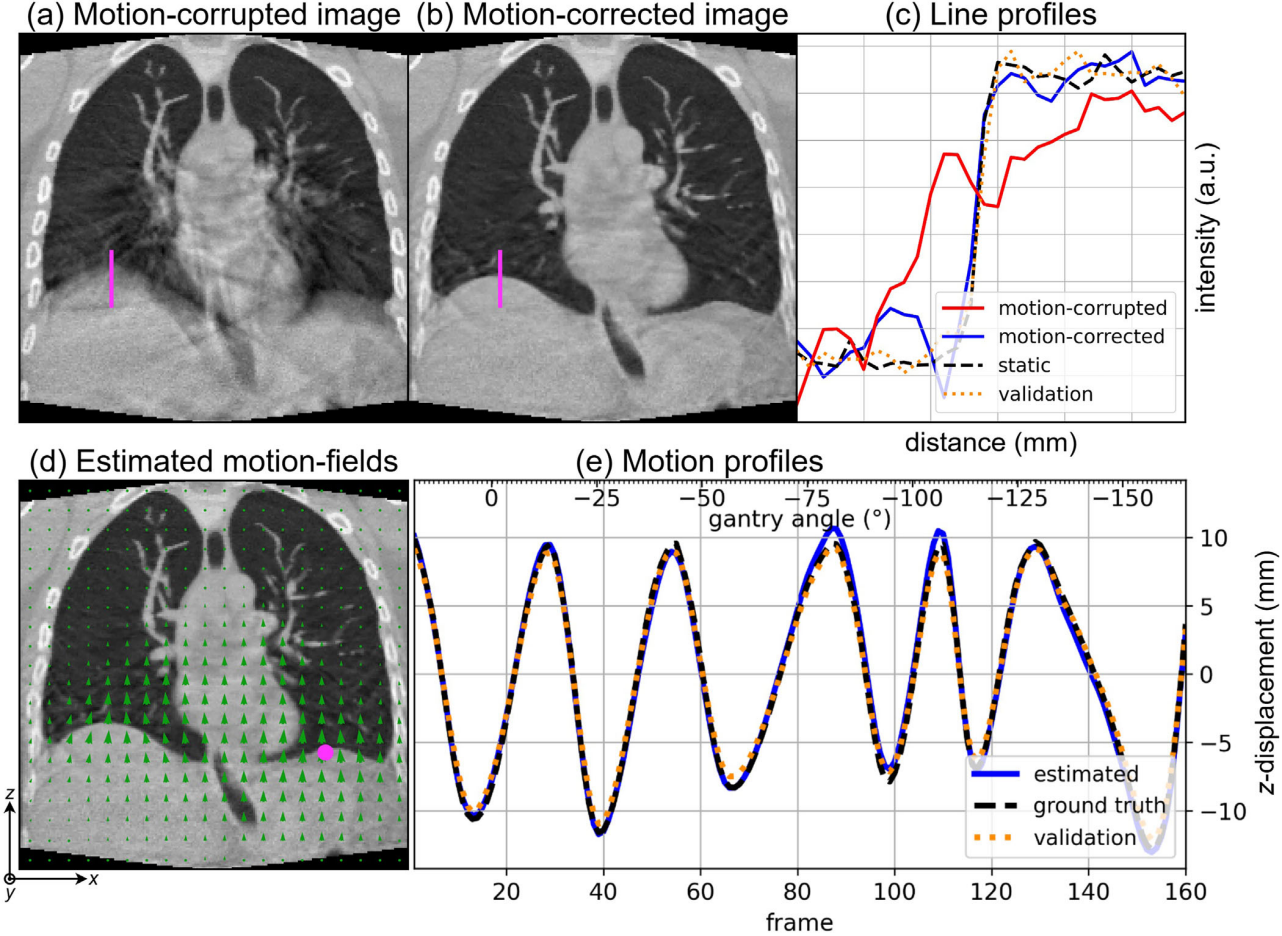
In silico (XCAT phantom): coronal slice of (a) the motion‐corrupted and (b) motion‐corrected images. (c) A line profile (magenta) through the lung–liver interface is taken of the motion‐corrupted, motion‐corrected, static and validation image reconstructions. (d) Coronal slice of the estimated motion‐fields (from reference to max‐exhale) imposed on the motion‐corrected image and (e) the motion profile in a voxel (magenta) tracing z‐displacement of the estimated, ground truth, and validation motion.

The motion‐corrected image of the in silico data shows improved image features (Figure 3). The motion‐corrupted image shows motion artifacts around the lung‐liver interface, heart and abdominal aorta (Figure 3a, 0.86 SSIM). The lung‐liver interface is visually sharp after applying motion correction on the in silico data, which was initially blurred due to the breathing motion of the XCAT simulation (Figure 3b, 0.90 SSIM). This motion artifact is reflected by its relatively flat line profile ($1/w_{\mathrm{ESF}}=0.12$ ${\mathrm{mm}}^{-1}$) with two steeper sections, indicating two contours (Figure 3c). The single steep line profile ($1/w_{\mathrm{ESF}}=0.40$ ${\mathrm{mm}}^{-1}$) of the final motion‐corrected image approximates the static image, indicating a sharp transition and reduction of motion artifacts. The image reconstruction with the motion‐field validation, where we use the ground truth XCAT image as the reference, shows that the applied alternations approach convergence (0.92 SSIM, $1/w_{\mathrm{ESF}}=0.37$ ${\mathrm{mm}}^{-1}$), indicating that the motion‐corrected image converges toward the ground truth.

The motion estimation captures the non‐rigid and irregular respiratory motion of the XCAT phantom (Figure 3d, Animated Figure 1 1). The non‐rigid motion is mostly estimated around the lung‐liver interface and other regions with high contrast. Little motion is estimated in the liver, and some ribs are moving too resulting in artifacts. The estimated motion profile in the lung–liver interface (Figure 3e) approximates the ground truth motion with a root mean squared error (RMSE) of [x:0.09,y:1.05,z:0.50] mm. The motion‐field validation profile shows that the applied alternations approach convergence, and has an RMSE of [x:0.09,y:1.17,z:0.32] mm w.r.t the ground truth.

**ANIMATED FIGURE 1:**
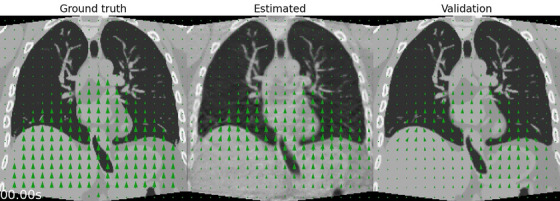
In silico (XCAT phantom): animation of the ground truth, estimated and validation motion. Motion‐fields are imposed and warp the ground truth and motion‐corrected images.

### 4.2 Phantom acquisition

**FIGURE 4:**
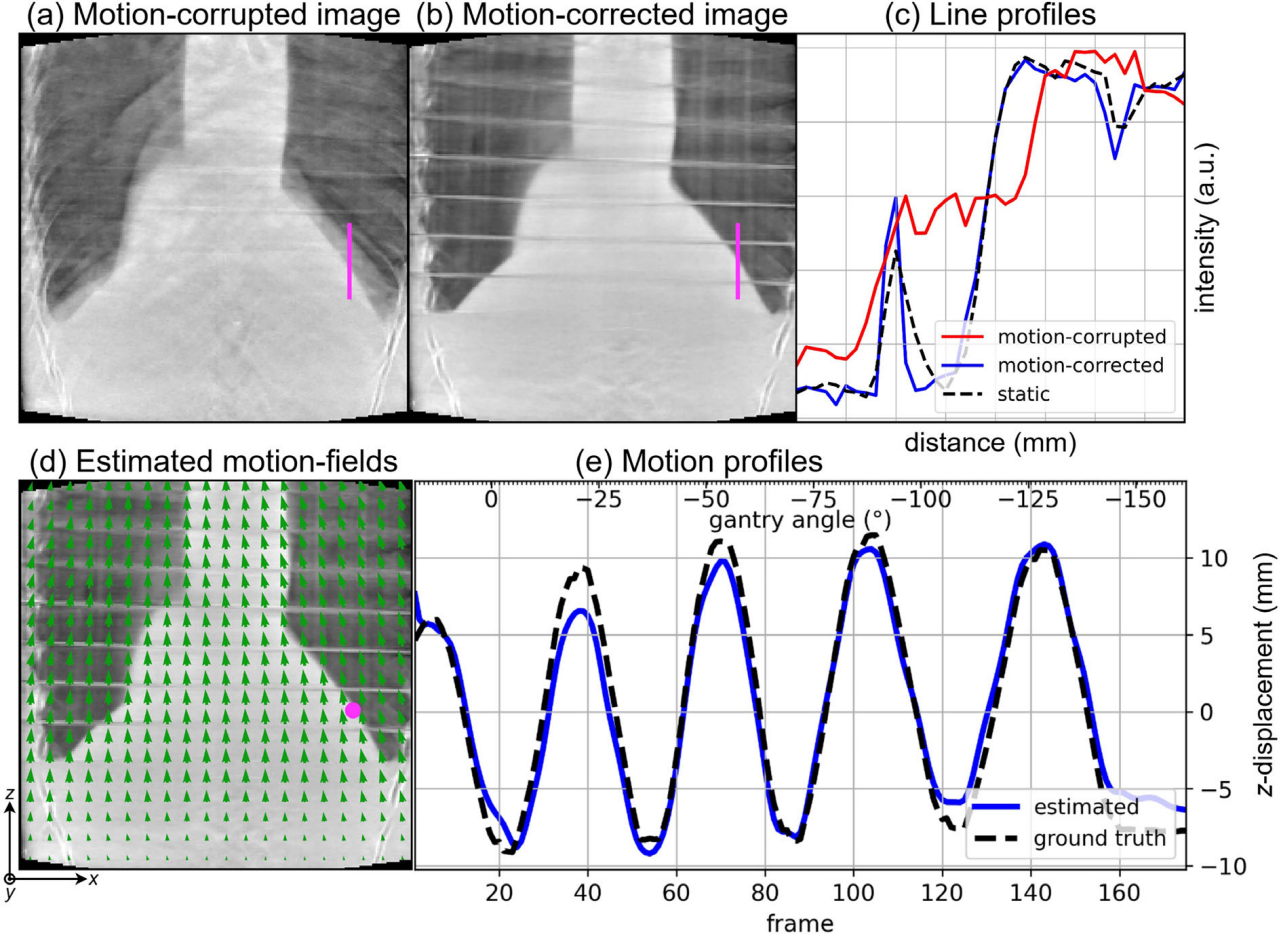
Alderson phantom acquisition: coronal slice of (a) the motion‐corrupted and (b) motion‐corrected images. (c) A line profile (magenta) through the lung–liver interface is taken of the motion‐corrupted, motion‐corrected and static images. (d) Coronal slice of the estimated motion‐fields (from reference to maximum amplitude) imposed on the motion‐corrected image and (e) the motion profile in a voxel (magenta) tracing z‐displacement of the estimated and ground truth motion.

The motion‐corrected image of the phantom acquisition shows reduced motion artifacts (Figure 4). The two contours of the lung–liver interface in the motion‐corrupted image (Figure 4a) have been corrected into one sharp contour. Moreover, the blurred gaps between the phantom slabs are more clearly defined after motion correction (Figure 4b). Note that gaps further from the center are less sharp due to cone‐beam artifacts, which we also observed in the static image reconstruction. The two steep line profiles of the final motion‐corrected image approximate the static reference image (Figure 4c), indicating recovery of image features. Note that the center gaps appear sharper as they are more parallel to the gantry.

The motion estimation captures the applied transverse rigid motion, where all deformation vectors point in the same direction (Figure 4d, Animated Figure 2, 1). Less motion is estimated near the homogeneous bottom region of the phantom. The largest deviation from ground truth is found around gantry angle $0^{\circ }$ (Figure 4e), which can be explained by the fact that at horizontal projections the motion information encoded in the 2D projections is overlapping for many organs. The estimated motion profile approximates the applied ground truth motion with an RMSE of [x:0.79,y:1.04,z:1.42] mm.

**ANIMATED FIGURE 2:**
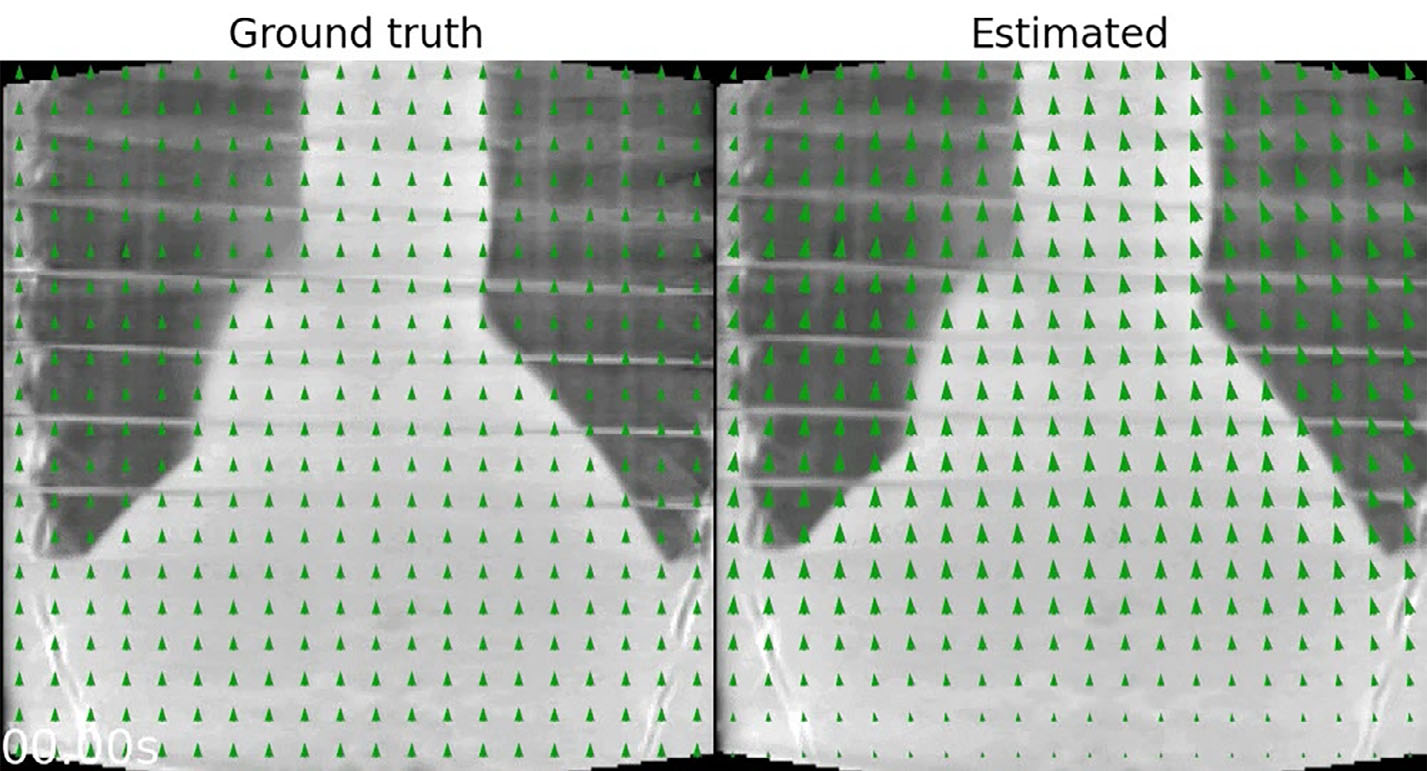
Alderson phantom acquisition: animation of the ground truth and estimated motion. Motion‐fields are imposed and warp the static and motion‐corrected images.

### 4.3 Clinical acquisitions

**FIGURE 5:**
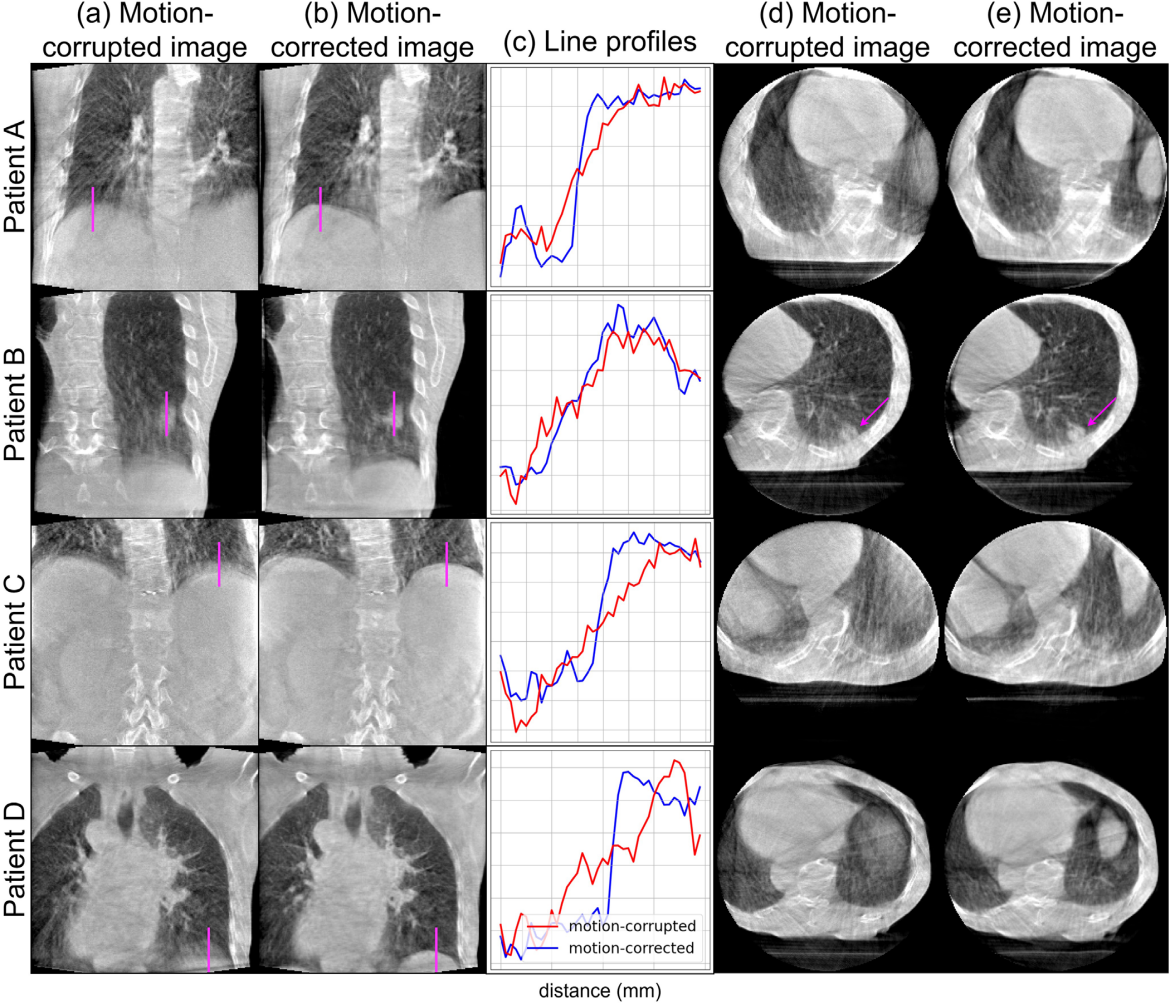
Clinical acquisition: coronal/axial slices of (a,d) the motion‐corrupted and (b,e) motion‐corrected images of patients A, B, C, and D. (c) Line profiles (magenta) through a tumor and the lung–liver interfaces are taken of the coronal motion‐corrupted and motion‐corrected images.

The motion‐corrected images of the clinical acquisitions show improved image features for all four patients (Figure 5). The initially blurred lung–liver interfaces of all patients (Figure 5a,d) have sharper appearances after motion correction in both coronal and axial views. Moreover, the outline of the lung tumor of patient B is more clearly defined (Figure 5b,e). The steeper line profiles show how the interfaces have sharpened visually, as opposed to the flat line profiles of the motion‐corrupted images (Figure 5c).

**FIGURE 6:**
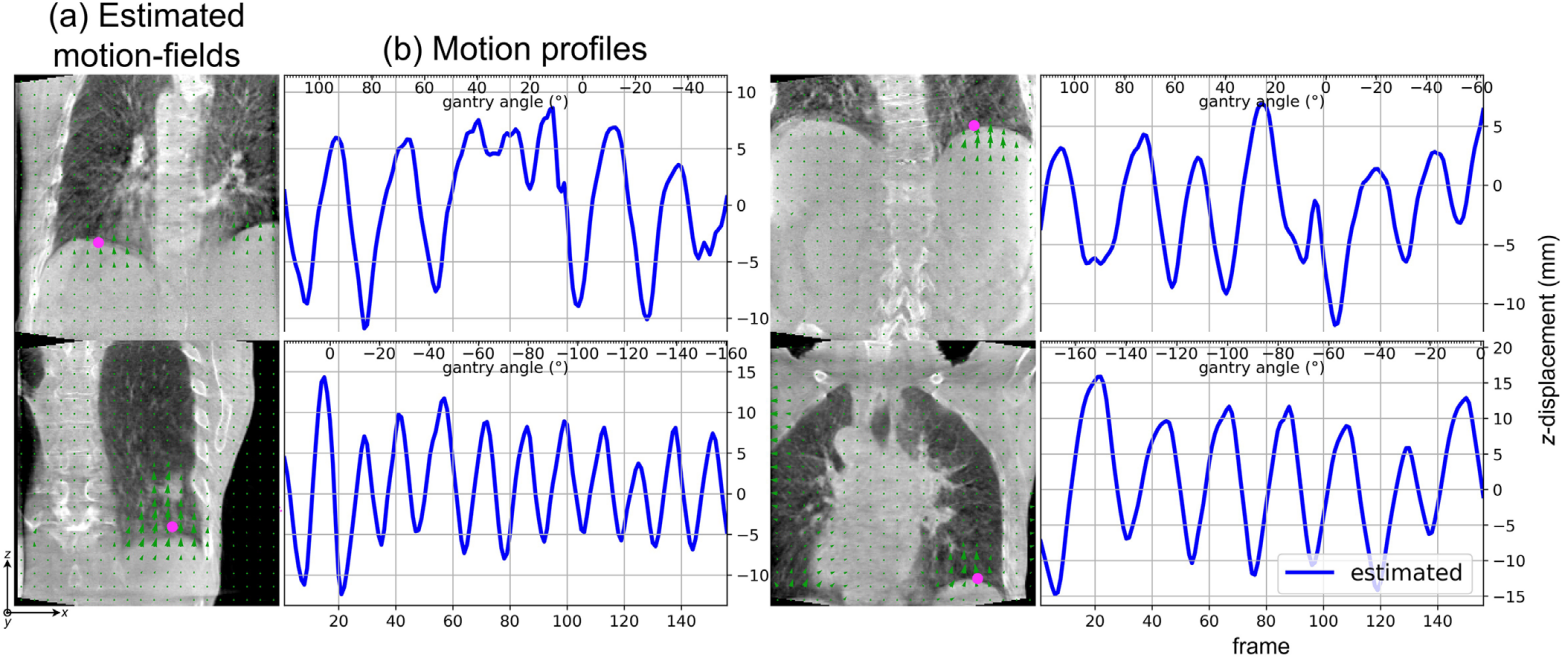
Clinical acquisition: (a) Coronal slices of the estimated motion‐fields (from reference to max‐exhale) imposed on the motion‐corrected images. (b) The motion profile in a voxel (magenta) tracing z‐displacement of the estimated motion.

The motion estimation captures the irregular and periodic non‐rigid respiratory motion of the patients (Figure 6a, Animated Figure 3, 1). Most of the motion is estimated around the lung–liver interface and patient B's tumor, which are regions with high contrast. The motion profiles near the lung–liver interface of patients B and D indicate regular breathing, while the breathing of patients A and C is more irregular (Figure 6b). Amplitudes of all estimated motion ranges between [5.2,30.6] mm (15.3 mm average) and periods between [2.0,6.0] s (3.6 s average), which are realistic and expected from real human breathing. We observe that patient A takes shorter breaths halfway through the scan. However, its motion profiles shows more jagged breathing around gantry angles of $0^{\circ }$. Moreover, we observe that motion is estimated near and outside the FOV, particularly for patient D. In short, the clinical data confirms that our gate‐less method is able to estimate irregular motion in a time‐resolved manner.

**ANIMATED FIGURE 3:**
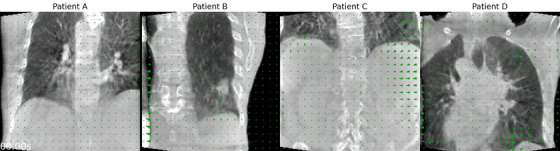
Clinical acquisition: animation of the estimated motion for all four patients. Motion‐fields are imposed and warp the motion‐corrected images.

### 4.4 Description of the animated figures

The animated figures are part of the main body of this work and can be found online.

### 4.5 Convergence

**FIGURE 7:**
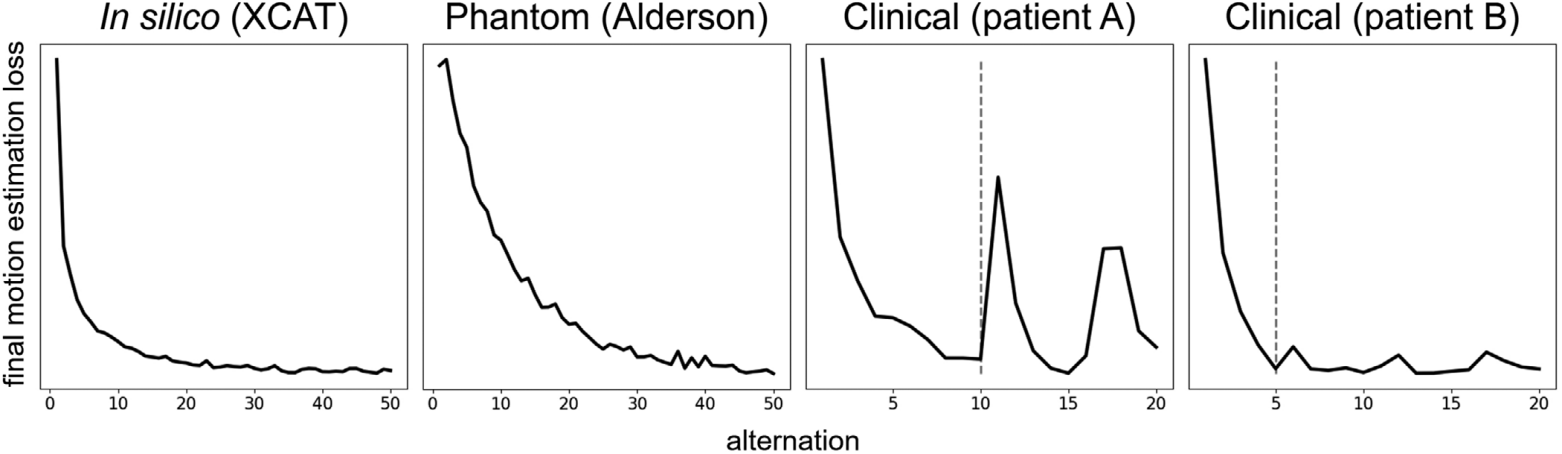
Convergence plots for the in silico, phantom and clinical (patients A and B) experiments. The vertical line indicates the converged alternation.

The motion estimation step converges for all three experiments (Figure 7). The final motion estimation loss of (7) decreases with each alternation. The convergence plot plateaus after about 50 alternations for the in silico and phantom experiments. The clinical experiments converged after 10,5,7, and 16 alternations for patients A, B, C, and D, respectively. We observe that applying further alternations increases the final loss and thus worsens the motion estimation and correction. Therefore, we stop the framework at the converged alternation.

## 5 DISCUSSION

The CBCT‐MOTUS framework estimates and corrects irregular and periodic non‐rigid motion with high temporal resolution (per‐projection temporal resolution of 182 ms), as shown in in silico simulations, phantom acquisitions and clinical data. The motion‐corrected images show improved image features for all experiments, such as deblurring. The motion is time‐resolved and represents real movement instead of averaged respiratory motion as no assumption on periodicity is used. Clinical gating typically requires prolonged, high‐dose 4D acquisitions to ensure robustness. In contrast, our method achieves reliable performance with shorter 3D scans at high per‐projection temporal resolution, underscoring its added clinical value.

Image features of all image reconstructions are improved for all experiments after performing motion correction, with an increase in SSIM and spatial resolution for the in silico experiment. The lung–liver interfaces are sharp and patient B's tumor is more clearly defined, which were initially blurred. The clinical relevance of the motion corrections is the decrease in uncertainty regarding motion artifacts in image reconstructions. The reference position appears to be near mid‐ventilation, as apparent from the motion profiles. If desired, a different reference position may be chosen prior to reconstruction.

We observe from the in silico and clinical experiments that motion is encoded in regions of high contrast. These regions are discernable in projection space and facilitate motion estimation. Therefore, motion is estimated around the lung–liver interface, while motion of the liver is not due its homogeneity. Estimating motion in homogeneous areas with no encoding power remains a challenge. The low‐rank motion model ensures a certain degree of smoothness in time and is able to extract respiratory signals in the temporal components. Future work includes applying this model to estimate complex, non‐compressible motion with decoupled spatial and temporal components (e.g., cardiac + respiratory), and other regions of the body (e.g., abdomen, extremities, brain/head). The TV spatial regularization on the Jacobian of the motion motion‐fields and cubic B‐spline parameterization ensure a certain degree of motion smoothness around the lung‐liver interface. However, non‐smooth motion, such as sliding motion, can be a major challenge for accurate motion estimation, as some ribs move along with the estimated motion. Future work includes matching the model complexity with the motion types.

Although image features are improved after one alternation, applying more alternations further improves both the motion estimation and correction gradually.^13^ The motion‐corrected images allow for better motion estimation and the estimated motion‐fields yield better motion‐corrected images. At convergence, the motion estimation no longer improves. As a consequence, the motion correction no longer improves either. We validated with the in silico experiment that using motion‐corrupted/corrected images as a surrogate for motion‐free reference images is effective. The phantom experiment corroborates this. The clinical experiments converged after a few alternations in terms of the loss function. Future work includes investigating the convergence properties, parameters, and criteria of the framework to fully automate it.

The results show that the CBCT‐MOTUS framework can estimate and correct motion despite not being optimized to correct for image‐degrading effects, such as photon scatter and ghosting. Ideally, changes in the measured signal intensities correspond only to patient motion. The framework can benefit from addressing these degradations by improving the signal model or using more advanced iterative reconstruction techniques^40^ rather than SIRT. Better image reconstruction quality can be achieved by, for example, performing photon scatter estimation, modeling the quantum noise and electronic noise that are intrinsic to the x‐ray detectors, and correcting for cone‐beam artifacts such as ghosting.

Performing one alternation takes 33 min with the settings from Table 1 and GPU‐acceleration, of which 25 min are for motion estimation and 8 min for motion correction. The motion estimation may be further accelerated by using other solvers for faster convergence such as Gauss‐Newton or L‐BFGS.^41^ Moreover, explicitly defining the gradients for faster convergence^13^ omits the use of PyTorch's autograd^32^ functionality. Deep learning could replace the motion estimation loop with networks that have the reference image and undersampled signals as input and motion‐fields as output, at the expense of having no guarantee of data fidelity. Moreover, the settings in Table 1 can be altered to tradeoff computation time and quality. Lower resolution images could be used for faster simulated projections for motion estimation.^7^

The motion estimation appears to be more challenging around gantry angles of $0^{\circ }$. The signal data is attenuated more when scanning the patient from the side and less information is visible than from the top. The quality of the motion estimation is thus angle‐dependent and sensitive toward parallel versus orthogonal motion with respect to the gantry angle. This is addressed by exploiting spatio‐temporal correlation in the low‐rank motion model and spatial motion‐field regularization. Another consideration is that motion outside the FOV (e.g., patient B's left lung) is not present in all the signal data and could affect motion estimation inside the FOV. This is handled by expanding the reconstruction domain to outside the FOV and estimate motion there for the relevant gantry angles. We stop at convergence to prevent overfitting due to extrapolation of motion and propagation of noise. The in silico and phantom experiments show, however, that the captured motion is quantitatively correct.

The CBCT‐MOTUS framework could be improved by performing parameter studies to determine optimal values for the parameters in Table 1, perhaps patient specific, and statistical analyses on more clinical acquisitions. Different convergence criterions looking at image features rather than motion optimization could be explored. Moreover, the impact of gantry rotation speed and other acquisition parameters could be investigated with different protocols. As mentioned previously, implementing photon scatter estimation and correction would improve the signal model, and therefore the motion estimation.

## 6 CONCLUSION

We have developed a gate‐less reconstruction method (CBCT‐MOTUS) for model‐based non‐rigid motion estimation and correction in CBCT imaging for irregular and periodic motion. The motion estimation is performed in projection space to achieve high temporal resolution (per‐projection temporal resolution of 182 ms) and estimate 3D motion‐fields per gantry angle. The correction of non‐rigid irregular and periodic motion is enabled by exploiting the smoothness and compressibility of motion using a low‐rank B‐spline motion model. The framework employs the motion‐corrupted images as the initial reference and signal data to alternate between motion estimation and motion‐corrected image reconstruction. We applied it to three proposed experiments, which all showed reduced motion artifacts and improved image features in the image reconstructions, and irregular and periodic motion patterns in the motion estimation.

## CONFLICTS OF INTEREST STATEMENT

This work was funded by the Dutch Cancer Society (KWF) with contributions from Philips and Tesla DC, and by the the Netherlands Organisation for Scientific Research (NWO). Casper Beijst, Rodrigo José Santo, Cornelis A.T. van den Berg, and Alessandro Sbrizzi, have a patent pending for the motion correction technique.

## Footnotes

1 The Animated Figures can be found online. See Section 4.4 for a description of all the animated figures.
